# Dose-Dependent Effect of Hydrogen Sulfide in Cyclophosphamide-Induced Hepatotoxicity in Rats

**DOI:** 10.5152/tjg.2023.22040

**Published:** 2023-06-01

**Authors:** Fikriye Yasemin Özatik, Orhan Özatik, Yasemin Tekşen, Havva Koçak, Neziha Senem Arı, Çiğdem Çengelli Ünel

**Affiliations:** 1 Department of Pharmacology, Kütahya Health Sciences University Faculty of Medicine, Kütahya, Turkey; 2 Department of Histology and Embryology, Kütahya Health Sciences University Faculty of Medicine, Kütahya, Turkey; 3 Department of Medical Biochemistry, Kütahya Health Sciences University Faculty of Medicine, Kütahya, Turkey; 4 Department of Pharmacology, Eskişehir Osmangazi University Faculty of Medicine, Eskişehir, Turkey

**Keywords:** Cyclophosphamide, hepatotoxicity, hydrogen sulfide

## Abstract

**Background:**

Cyclophosphamide is a commonly used anticancer and immunosuppressive agent; however, hepatotoxicity is one of its severe toxicities. Hydrogen sulfide is a gaseous signaling molecule that plays crucial regulatory roles in various physiological functions. This study aimed to evaluate the hepatoprotective effect of hydrogen sulfide against cyclophosphamide-induced hepatic damage in rats.

**Methods:**

Hepatotoxicity was induced by the single intraperitoneal administration of cyclophosphamide (200 mg/kg). Sprague–Dawley rats were treated by hydrogen sulfide donor, sodium hydrosulfide (25, 50, and 100 µmol/kg, intraperitoneal) 7 days before and 7 days after the administration of a single intraperitoneal injection of cyclophosphamide (200 mg/kg). Cyclophosphamide-induced hepatotoxicity was evaluated by serum and tissue biochemical and histopathological assessments. The levels of hydrogen sulfide, nitric oxide, cyclic guanosine monophosphate, interleukin 6, and interleukin 10 in liver homogenates were also determined by ELISA. One-way analysis of variance and Kruskal–Wallis tests were used as statistical analyses.

**Results:**

Cyclophosphamide increased liver function enzymes (alanine aminotransferase and aspartate aminotransferase), immunoreactivity to caspase-3 and Apaf-1, and proinflammatory cytokines. Cyclophosphamide also induced histopathological alterations including pycnotic nucleus with eosinophilic cytoplasm, increased sinusoidal dilatation, congestion, and edema. Hydrogen sulfide co-treatment significantly reduced cyclophosphamide-induced inflammation, histological alterations, and apoptosis in the liver. 50 mg/kg sodium hydrosulfide was more effective against cyclophosphamide-induced hepatotoxicity.

**Conclusion:**

In conclusion, hydrogen sulfide with its anti-inflammatory and anti-apoptotic effects seems to be beneficial as an adjunct to cyclophosphamide treatment to reduce cyclophosphamide-induced hepatotoxicity and thereby can be suggested as a promising agent to increase the therapeutic efficacy of cyclophosphamide.

**Main Points**

- Exogenous hydrogen sulfide (H_2_S) seems to ameliorate hepatic damage induced by cyclophosphamide (CP).
- The most effective dose range is between 25 and 100 µmol/kg and 50 µmol/kg H_2_S donor sodium hydrosulfide was more effective against CP-induced hepatotoxicity.
- Hydrogen sulfide may be useful in preventing CP-induced hepatotoxicity and can be a promising agent to increase the therapeutic efficacy of CP.

## Introduction

Cyclophosphamide (CP), an oxazophosphorin derivative, is used in the treatment of various nonneoplastic and neoplastic diseases for more than 5 decades.^1^ Cyclophosphamide is widely metabolized via cytochrome P450 enzymes in the liver. As a result of its metabolism, 2 active molecules are formed, phosphoramide mustard and acrolein that cause tissue damage via oxidative stress.^2^ Since CP is metabolized by the liver, the liver is the primary target for drug-related tissue damage. Particularly, acrolein is primarily responsible for the hepatotoxicity of CP that causes tissue injury via apoptosis or necrosis in the presence of GSH depletion. Therefore, liver cells fail to protect themselves against the toxicity of oxygen radicals.^3^ An increase in lipid peroxidation and reactive oxygen species (ROS) causes damage to mitochondria and impairs cellular respiration.^4^ Free radicals are also known to be involved in the etiology and pathophysiology of inflammation. Besides cell damage, ROS activates several signaling pathways (i.e., the nuclear transcription factor kappa-B [NF-kB]) that cause inflammation and also cell death.^4,5^ Therefore, finding a relatively safe agent to protect against CP-induced hepatotoxicity is a critical issue in the clinical use of CP.

Hydrogen sulfide (H_2_S) is a colorless and water-soluble gas with a distinctive malodor of rotten eggs. Hydrogen sulfide is mainly generated from l-cysteine and l -homocysteine by cystathionine у-lyase (CSE) and cystathionine β synthase (CBS) enzymes.^6^ 3-Mercaptopurivate sulfurtransferase (3-MST) plays a role with cysteine aminotransferase (CAT) in the synthesis of H_2_S from l-cysteine in the presence of α ketoglutarate in the liver.^7^ Hydrogen sulfide is recognized as one of the main gaseous signaling molecules that play crucial regulatory roles in various physiological events including angiogenesis, vasodilatation, and neuronal activity.^6^ The liver plays an essential role in lipid and glucose metabolism, antioxidant action, and xenobiotic metabolism. It is also important for the generation and clearance of H_2_S.^8^ Hepatic H_2_S plays a role in mitochondrial biogenesis and bioenergetics, insulin sensitivity, lipoprotein synthesis, and glucose metabolism. In addition, H_2_S also is involved in the pathogenesis and treatment of several liver diseases including cirrhosis, liver carcinoma, hepatic ischemia/reperfusion injury, and non-alcoholic liver disease.^6^ A recent study demonstrated that uranium poisoning reduced the formation of endogenous H_2_S in hepatic homogenates, whereas NaHS could diminish acute uranium-induced hepatotoxicity in rats via antioxidant and antiapoptotic signaling pathways.^9^ Furthermore, H_2_S treatment was also shown to partially attenuate acetaminophen hepatotoxicity in mice via its antioxidative and anti-inflammatory actions.^10^

In this research, we hypothesized that H_2_S donor NaHS might be effective in the treatment of CP-induced hepatotoxicity. We aimed to evaluate the possible therapeutic and/or protective dose-dependent actions of H_2_S against CP-induced hepatotoxicity in rats.

## Materials and Methods

### Experimental Animals

The experimental procedures were conducted in the Experimental Animal Breeding Research and Application Center and Department of Medical Pharmacology at Kütahya Health Sciences University. All actions related to animals were carried out in accordance with national and international regulations on animal experiments (ethical approval number: 2019.01.06).

In this study, 40 male Sprague–Dawley rats, weighing 240-280 g, were used. The rats were housed in a temperature-controlled (24 ± 2°C) room with relative humidity (55% ± 15%) and standard 12 hours light/12 hours dark cycles. Rats were fed with standard food and water ad libitum. All rats were acclimatized for 1 week before the experiments. The group size of n = 8 animals for the experiments was determined by sample size estimation using G*Power (v3.1)^11^ to detect the size effect in a post hoc test with type 1 and 2 error rates of 5 and 20%, respectively.

Rats were randomly allocated into five groups as follows (n = 8):

**Control group:** Animals received 0.2 mL of normal saline i.p for 15 days.
**CP group:** Animals received 0.2 mL saline i.p for 7 consecutive days, before and after CP (Eczacibasi, Turkey) injection (200 mg/kg ip).^12,13^
**NaHS25 group:** Animals received NaHS (Sigma, USA) 25 μmol/kg i.p for 7 consecutive days, before and after CP injection (200 mg/kg ip).
**NaHS50 group:** Animals received NaHS 50 μmol/kg i.p for 7 consecutive days, before and after CP injection (200 mg/kg ip).
**NaHS100 group:** Animals received NaHS 100 μmol/kg i.p for 7 consecutive days, before and after CP injection (200 mg/kg ip).^14^

Twenty-four hours following the last dose of the treatment, all animals were weighed and intracardiac blood samples were collected under ketamine (70 mg/kg ip, Ketalar, Pfizer, Turkey) and xylazine (10 mg/kg ip, Alfazyne, Atafen, Turkey) anesthesia. Serum was centrifugated for 20 minutes at 1000 *g* and stored at −20°C until biochemical analysis. Then the animals were euthanized by an overdose of anesthesia. The livers of the rats were isolated and divided into 2 equal parts for histopathological examination and for the preparation of biochemical homogenates.

### Biochemical Analysis

The biomarker enzymes for liver functions such as aspartate aminotransferase (AST) and alanine aminotransferase (ALT) concentrations in serum samples were measured with an automatic analyzer (Beckman Coulter, AU680 Miami, Fla, USA). Freshly isolated serum was mixed with a working solution and the conversion of NADH to NAD was measured spectrophotometrically at 340 nm. The unit of enzyme activity was expressed as IU/L.

### Histopathological Examination

The liver tissues were immediately fixed in 10% neutral buffered formalin. Tissues were then embedded in paraffin blocks, sectioned, and stained with hematoxylin and eosin (H&E). The immunoreactivity of APAF-1 and caspase-3 was examined to show apoptotic cells under light microscopy.

A point count method was used to assess the severity of hepatic damage. The degree of damage was scored as follows.^15^

**Grade 0:** No or minimal damage;
**Grade 1:** Nuclear pycnosis with mild damage;
**Grade 2:** Expanded nuclear pycnosis with mild damage, cytoplasmic hypereosinophilia, and loss of cell borders, and;
**Grade 3:** Neutrophil infiltration with extensive damage.

### Investigations on Liver Homogenates

#### Preparation of Liver Homogenates

Liver tissues were homogenized in 5% Triton X-100 and phosphate-buffered saline (PBS) (1:1). They were centrifuged at 1000 *g* for 10 minutes and the supernatant was used in biochemical analysis. The levels of hydrogen sulfide, nitric oxide, cyclic guanosine monophosphate, interleukin 6, and interleukin 10, the levels of H2S (Sunredbio, Shanghai, China), nitric oxide (NO) (R&D Systems, Minneapolis, USA), cGMP (Elabscience, Huston, Texas, USA), IL-6 (Elabscience, USA), and IL-10 (Elabscience, USA) were analyzed in liver homogenates by using ELISA commercial kits according to manufacturer’s instructions.

## Statistical Analysis

Data were analyzed using Statistical Package for Social Sciences 21.0 software program (IBM Corp.; Armonk, NY, USA). The data were expressed as mean ± standard error of mean (SEM). A one-way analysis of variance (ANOVA) test followed by Dunnett as a post hoc test was used for biochemical analysis. The Kruskal–Wallis followed by Dunn method as a post hoc test was used to compare the histopathological data. *P*  <  .05 was accepted as significant.

## Results

### Effects of Hydrogen Sulfide on Biochemical Analysis

**Figure 1.**
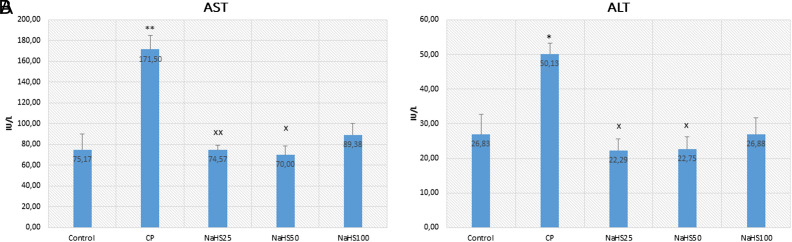
Serum AST and ALT values. A: AST; B: ALT. Data are given as mean ± SEM. *Different from control ; **P < 0.01; ^x^Different from CP; ^x^ *P* < 0.05; ^xx^ *P* < 0.01; ANOVA (n = 8). ANOVA, analysis of variance; C, control; CP, cyclophosphamide; NaHS, sodium hydrosulfide; SEM, standard error of mean.

Aspartate aminotransferase and ALT are the main enzymes found in the liver. The increase in these enzymes indicates damage and inflammation in liver cells. In the study, AST values increased in the CP group (*P* < .01) (Figure 1A). This result shows that CP caused liver damage and inflammation in the liver. However, a significant reduction was seen in the AST levels of NaHS treatment groups compared to the CP group. This decrease is more significant especially in the NaHS50 group. This value was higher in the NaHS100 group than in the other NaHS-treated groups. 50 µmol/kg NaHS seems more effective and able to ameliorate the liver damage induced by CP. Similar results were obtained in ALT values as in the case of AST values. Alanine aminotransferase also significantly increased in the CP group compared to the control group (*P* < .05). There is a significant reduction in NaHS25 and NaHS50 groups compared to the CP group (*P* < .05) (Figure 1B).

### Effects of Hydrogen Sulfide on Histopathological Evaluation

Normal histological findings were obtained in the control group. Pycnotic nucleus with eosinophilic cytoplasm, increase in sinusoidal dilatation, sinusoidal congestion, and edema were observed in many parts of the parenchyma in sections of the CP group. In addition, mononuclear cell infiltrations were also observed. The hepatocytes of the NAHS25 group showed less pycnotic nuclei with eosinophilic cytoplasm in the parenchyma, wider sinusoidal dilatation, sinusoidal congestion, and edema than the hepatocytes of the CP group.

**Figure 2.**
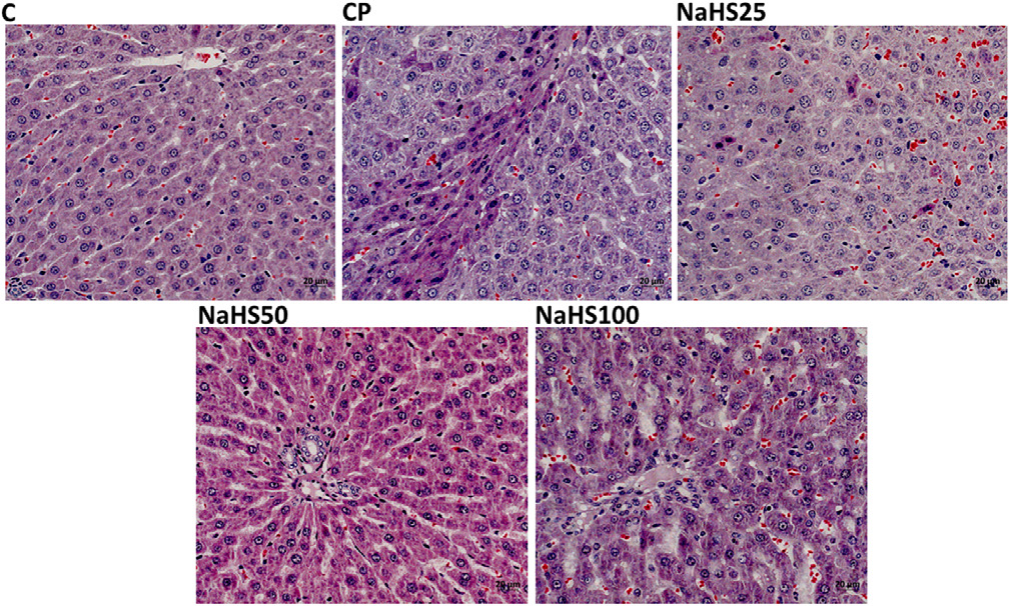
Representative images of H&E staining in liver tissues (×40). C, control; CP, cyclophosphamide; H&E, hematoxylin and eosin; NaHS, sodium hydrosulfide.

There was no histopathological abnormality in the tissue sections of the NaHS50 group, except for an increase in sinusoidal dilatation in the parenchyma. In the NaHS100 group, an increase in sinusoidal dilatation, sinusoidal congestion, and edema were observed at similar levels to that of the CP group. Pycnotic nuclei with eosinophilic cytoplasm were observed in partial sections of tissues (Figure 2).

**Figure 3.**
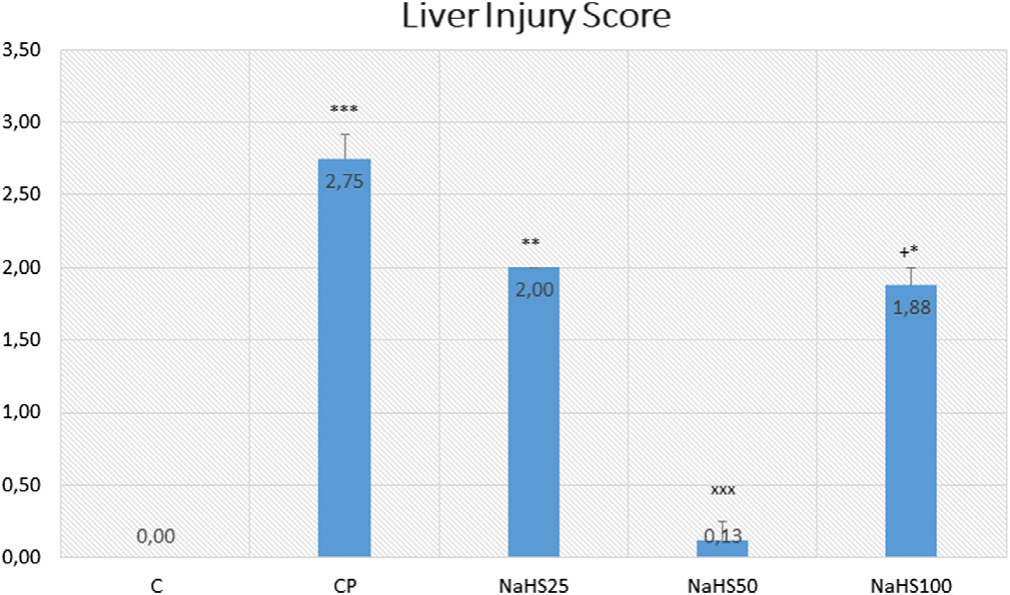
Effects of NaHS treatment on histological parameters in rats with CP toxicity. Data are given as mean ± SEM. *Different from control; ***P < .001; **P < .01; *P < .5; ^+^Different from NaHS50; ^+^ *P* < .5 ANOVA (n = 8). ANOVA, analysis of variance; C, control; CP, cyclophosphamide; NaHS; sodium hydrosulfide; SEM, standard error of mean.

There was no hepatic damage observed in the control group. However, hepatic damage increased in the CP group compared to the control group (*P* < .001) and decreased in the NaHS treatment groups. Similar results were obtained in NaHS50 and control groups (Figure 3).

The marked hepatic damage was observed in the NaHS100 group (*P* < .05). Thus, 50 µmol/kg NaHS was found to be the most effective dose in the study.

**Figure 4.**
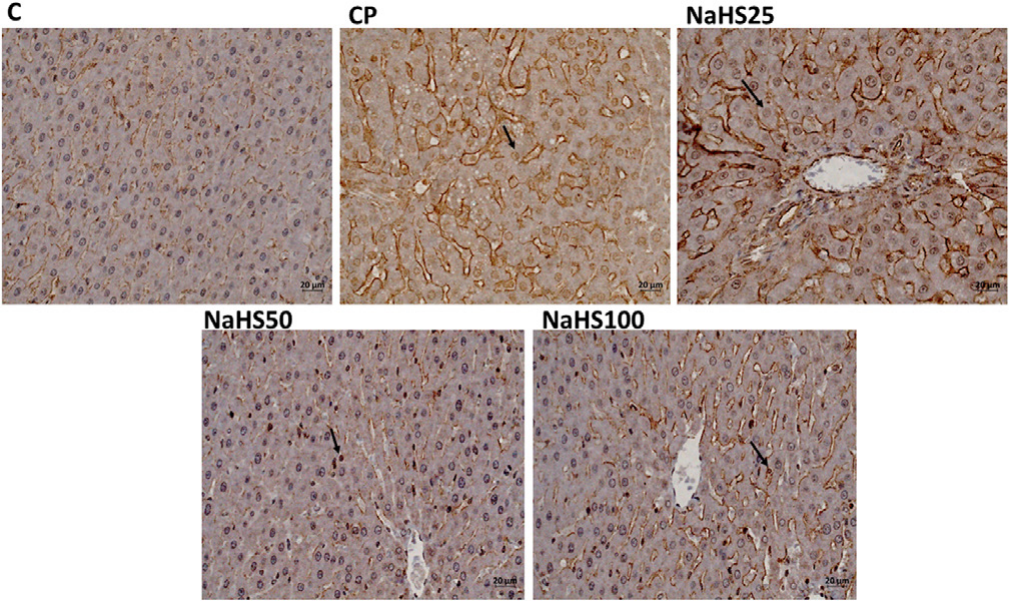
Representative images of caspase-3 staining in liver tissues (×40). C, control; CP, cyclophosphamide; NaHS, sodium hydrosulfide.

**Figure 5.**
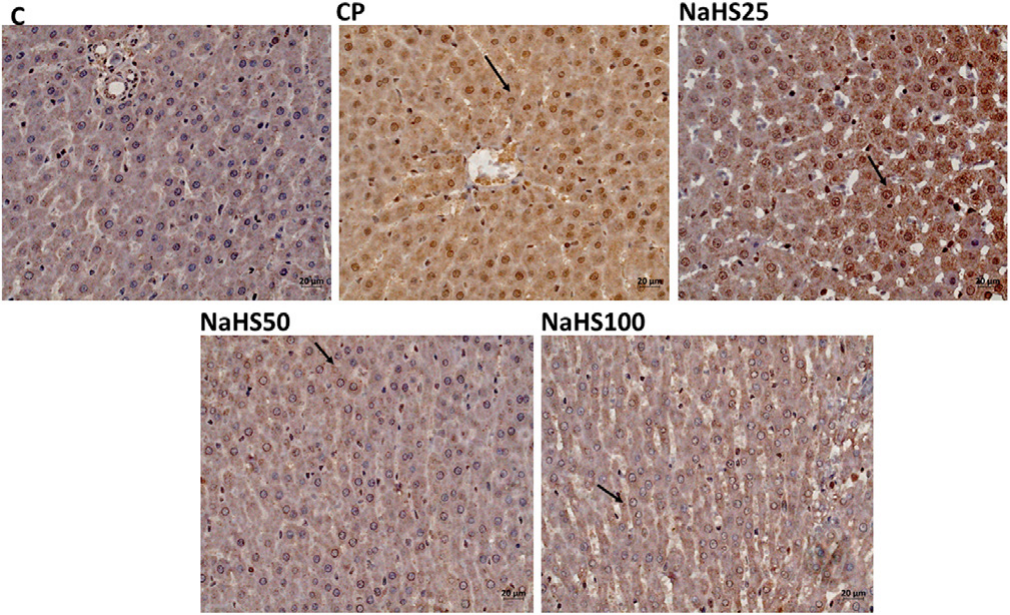
Representative images of APAF-1 staining in liver tissues (×40). C, control; CP, cyclophosphamide; NaHS, sodium hydrosulfide.

**Figure 6.**
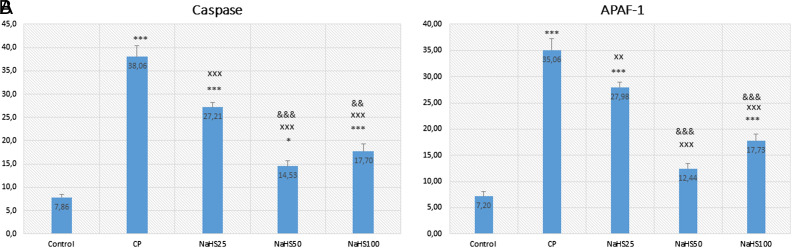
Effects of NaHS treatment on immunohistochemical parameters in rats with CP toxicity. A: caspase-3; B: Apaf-1. Data are given as mean ± SEM. *Different from control; *P < 0.05; ***P < 0.001; ^x^Different from CP; ^xx^ *P* < 0.01; ^xxx^ *P* < 0.001; ^&^Different from NaHS25; ^&&^ *P* < 0.01; ^&&&^ *P* < 0.001 ANOVA (*n* = 8). ANOVA, analysis of variance; C, control; CP, cyclophosphamide; NaHS, sodium hydrosulfide; SEM, standard error of mean.

Apoptotic index analysis revealed that both caspase-3 and APAF-1 immunoreactivity were significantly higher in the CP group compared to the control group (*P* < .001) (Figures 4, 5, 6A and 6B). However, there is a decrease in the NaHS treatment groups. Especially the decrease in the NaHS50 group is significant in both immunohistochemical stainings. This result also indicates that NaHS was able to attenuate apoptosis resulting from CP treatment at a dose of 50 µmol/kg (*P* < .001) (Figures 6A and 6B).

### The Effects of Hydrogen Sulfide in the Evaluations of Hydrogen Sulfide, Nitric Oxide, Cyclic Guanosine Monophosphate, Interleukin-6, and Interleukin-10 in Liver Homogenates

Interleukin (IL)-6 acts as a pro-inflammatory cytokine and an anti-inflammatory myokine. In the study, IL-6 values increased in the CP group compared to the control (*P* < .001) (Table 1). The IL-6 levels decreased in the NaHS treatment groups and the highest decrease was seen in the NaHS50 group. Similar results were obtained in IL10 levels. It is noticeable that the values of both parameters increased in the NaHS100 group (*P* < .001). There was a statistically significant decrease in cGMP values of the CP group compared to the control group. An increase was observed in the NaHS treatment groups and the highest value was observed in the NaHS50 group (*P* < .01). The levels of NO decreased in the CP group compared to the control group while increased in the NaHS50 group when compared to the CP group. Thus, a dose of 50 μmol/kg NaHS was able to keep NO levels under control.

When H_2_S values were compared, a decrease was observed in the CP group and this level began to rise in the NaHS treatment groups and the highest value was observed in the NaHS50 group (Table 1).

## Discussion

In the study, we investigated the possible therapeutic and/or protective dose-dependent effects of H_2_S against CP-induced hepatotoxicity in rats. Sodium hydrosulfide, an H_2_S donor, was used in 3 different doses to determine the most effective dose of H_2_S and to investigate if it has a dose-dependent action.

Drug-induced liver injury is the leading cause of acute liver failure worldwide. Cyclophosphamide is one of the nitrogen mustard group of alkylating chemotherapeutic agents which is involved in various physiological functions such as immune regulation and antitumor angiogenesis. Cyclophosphamide is a chemotherapeutic agent in the treatment of various malignancies including lymphoma, leukemia, breast cancer, and small-cell lung carcinomas. Furthermore, it is also used to suppress the immune system prior to bone marrow transplantation.^16,17^ In a study, it was reported that approximately 16% of patients who had hematopoietic cell transplantation suffered from hepatic sinusoidal obstruction syndrome (HSOS).^18^ High doses of CP may trigger acute hepatotoxic effects by inducing inflammation and oxidative stress in the liver.^19^ Cyclophosphamide is metabolized to phosphoramide mustard and acrolein in hepatocytes via cytochrome P450 enzymes. In a previous study, while phosphoramide mustard was able to demonstrate antineoplastic activity, acrolein contributed to hepatotoxicity.^20^ Thus, hepatotoxicity is one of the biggest problems in CP use. Our results show that the administration of a single and high dose of CP caused acute hepatotoxicity.

In this study, we aimed to evaluate the effects of NaHS (25-100 µmol/kg), an H_2_S donor agent, against CP-induced hepatotoxicity. The striking result of the study is that 50 µmol/kg NaHS has an impact on all parameters. A marked elevation in the levels of aminotransferases (ALT and AST) in CP-treated rats was a significant indicator of a hepatic injury. Similarly, in a study by Omar et al^21^ investigating the effects of tangeretin, a flavonoid, on cisplatin-induced hepatic damage, an increase was also found in ALT and AST levels, especially in the cisplatin group. Cuce et al^22^ investigated the chemoprotective effect of vitamin E on CP-induced hepatotoxicity and they found increased levels of AST and ALT in the CP group. Cyclophosphamide causes hepatotoxicity by inducing oxidative stress in the liver. Hepatotoxicity may occur even after low doses of CP.^23^ Serum levels of ALT and AST are related to the severity of CP-induced hepatotoxicity.^24^ In a previous study investigating the effects of H_2_S in acute liver injury induced by crushing the hindlimbs of rats, AST and ALT values increased with trauma, and the level of H_2_S in serum decreased. In addition, they proposed that a decrease in H_2_S levels may contribute to acute liver injury.^25^ In our study, we have seen a reduction in AST and ALT values in H_2_S donor-treated groups and a decrease in H_2_S values in the CP-treated group. Thus, we have similar results with the study suggesting that the treatment with a NaHS donor was able to attenuate the hepatic injury.^21^ In our study, cyclophosphamide treatment caused liver damage and a reduction in H_2_S tissue levels. In addition, AST and ALT values increased in the CP group and decreased in the NaHS treatment groups.

In histological evaluation, H&E, caspase, and Apaf-1 dyes were used to determine the severity of hepatic damage and apoptosis. Cyclophosphamide group significantly has higher damage scores in the liver. However, NaHS treatment (50 µmol/kg) seemed to improve the histology very close to that of the control group. In a study by Liu et al^26^ they found that exogenous NaHS at high doses (5 mg/kg) alleviated the hepatic damage induced by paraquat. The histologic examination with H&E staining revealed similar results to our study. The immunoreactivity to caspase-3 and Apaf-1 staining was determined as higher in the CP group and lower in the NaHS treatment groups. It was reported that the H_2_S has a crucial role in the CBS/CSE system in the regulation of cell metabolism, inflammation, and oxidative stress. Exogenous H_2_S supplementation was shown to attenuate apoptosis by hindering the MAPK signaling pathway. Lin et al^27^ reported that H_2_S can protect umbilical vein endothelial cells against high glucose-induced damage. The same researchers tried to predict CSE and CBS expressions in different organ types and found that the highest expression level of CSE/CBS was found in the liver. In addition, they investigated whether H_2_S ameliorated N-Asetil-P-aminofenol (APAP)-induced acute liver injury and proved that exogenous H_2_S ameliorated APAP-induced liver injury *in vivo*. They reported that H_2_S attenuated APAP-induced apoptosis via JNK/MAPK signaling pathway in hepatocytes^28^ similar to our study. 

In our study, H_2_S levels in liver tissues decreased in the CP group while a non-significant increase was found in the H_2_S levels of NaHS groups. The increase of this level in the NaHS50 group was very close to that of the control group. Sodium hydrosulfide 50 µmol/kg dose seems to provide a therapeutic effect on CP-induced liver damage. This result was also supported by immunohistochemical results. As with the 2 different gasotransmitters NO and CO, H_2_S induces a concentration-dependent biphasic effect, cytoprotective and cytotoxic.^29^ Ischemia–reperfusion (I/R) is a common method in major surgery and liver organ transplantation.^30^ Hydrogen sulfide protects the liver against total/hepatic I/R damage and dysfunction.^31^ Kang et al^32^ predicted that NaHS might have protective effects on liver tissue after hepatic ischemia-reperfusion injury. They attributed this effect to its anti-inflammatory activity by hindering the release of proinflammatory mediators and neutrophil accumulation, to its antioxidative activity by diminishing lipid peroxidation and possibly to the regulation of down-caspase-3, TNFα, and Fas/FasL. Another study also showed that uranium intoxication reduced the formation of endogenous H_2_S levels in hepatic homogenates, whereas NaHS was able to decrease uranium-induced acute hepatotoxicity with antioxidant and antiapoptotic signaling pathways in rats.^33^

In our study, while NO values were reduced in the CP group, they were higher in the NaHS-treated groups. Hydrogen sulfide and NO share several similar biological features in common. They are both cell-permeable gases under physiological conditions and are synthesized from amino acids including cysteine and arginine in some mammalian cell types. Whiteman et al^34^ showed that these 2 similar gases interact with each other. In addition, in the sepsis model by using lipopolysaccharide (LPS), the upregulation of both NO and H_2_S biosynthesis in liver homogenates was abolished following LPS injection in rats. This was explained by the inhibition of transduction via NF-kB.^35^ In addition, H_2_S can regulate angiogenesis through selective potentiation of other molecules such as NO and CO.^36^ Recently, Raina et al^37^ investigated the effects of endogeneous and exogeneous H_2_S on toll-like receptors (TLRs) – mediated inflammatory response and apoptosis in the CP-induced hepatotoxicity model. In this study, NaHS, an H_2_S donor agent, at a dose of 100 µmol/kg, and dl-propargylglycine (PAG), an H_2_S blocker, at a dose of 30 mg/kg were administered to rats. As a result of this study, exogeneously administered NaHS was able to protect hepatocytes against CP-induced hepatotoxicity mediated by the TLRs/JNK/NF-kB pathway. Nitric oxide levels were found to be 1.65 times higher in CP-treated rats compared to the control group. Sodium hydrosulfide treatment led to a significant decrease in NO levels. This result is not compatible with the NO levels in our study. Increased NO levels were proposed to be associated with increased oxidative stress caused by CP treatment.^37^

Some studies have shown that H_2_S stimulates angiogenesis through some enzymatic and non-enzymatic pathways.^38,39^ Bucci et al^40^ showed that H_2_S increased cGMP in smooth muscle cells by inhibiting phosphodiesterase activity. This effect of H_2_S-induced endothelial proliferation and migration under hypoxic conditions.^41^ In this study, we observed that cGMP levels decreased in the CP group compared to the control group, and there was a slight increase in the NaHS treatment groups.

In our study, we detected higher levels of the pro-inflammatory cytokine, IL-6 in the tissue homogenates of the CP group. Interestingly, although the IL-6 level was decreased to control levels, especially in the NaHS50 group, this level increased in the NaHS100 group. Thus, NaHS was found to exert toxic effects at higher doses. In a previous study of methotrexate (MTX)-induced hepatotoxicity higher levels of IL-6 were found in the MTX group; however, a significant reduction was found in the H_2_S+MTX group.^42^ In addition, H_2_S significantly reduced STAT3 expression in liver tissue which plays an important role in the pathogenesis of acute phase response in hepatic damage to minimize histopathological damage.^42,43^ In this study, H_2_S was reported to reduce MTX-induced hepatotoxicity and provide hepatoprotection with its antioxidant, anti-inflammatory, and anti-apoptotic effects via the suppression of the IL-6/STAT3 signaling pathway. The nuclear transcription factor kappa-B is one of the important transcriptional factors involved in mediating CP-induced cytotoxicity. In a previous study, NaHS was also shown to cause a reduction in TRL2/4, NF-kB, and TNF-α levels.^37^ In another study, CP led to a significant elevation in the levels of proinflammatory cytokines including TNF-α and IL-6 which are involved in the mechanism of tissue damage.^44^ In the study, NaHS was also able to reduce IL-6 levels which were elevated by CP administration. 

Interleukin (IL)-10 is an anti-inflammatory cytokine that has a very important role in preventing inflammatory and autoimmune pathologies. In our study, the higher levels of IL-10 increased in the CP group. Younis et al^45^ investigated the possible protective effects of silymarin and the role of endogenous H_2_S in rats with insulin resistance and hepatic ischemia-reperfusion injury (HIR). Unlikely, the researchers found a decrease in IL-10 values in the HIR group which is another inflammatory experimental model. Our result can be considered a response to inflammation.

Another remarkable result in our study is that there was a deviation from control levels in all parameters in the group given 100 µmol/kg NaHS. Thus, H_2_S seems to be toxic at higher doses. In previous studies, ameliorative effects of NaHS were observed at a dose of 50 µmol/kg.^42^ In our study, 50 µmol/kg NaHS was also able to ameliorate CP-induced hepatic damage similar to the control group. We also previously found that 50 µmol/kg NaHS induced a protective effect against kidney, bladder, and testis toxicity.^46,47^

The study needs to be supported with more parameters. We also examined the dose ranges of H_2_S in different organs. We got the same results in all of them. In this study, we could not perform cell culture studies due to budget constraints. However, in the next phase of the study, these dose ranges should be examined by performing in vitro experiments. 

## Conclusion

In conclusion, exogenous H_2_S seems to ameliorate CP-induced hepatic injury via its anti-inflammatory and anti-apoptotic effects. Sodium hydrosulfide, at doses of 25-100 μmol/kg, was used in previous studies and toxic effects may be seen above 100 μmol/kg. In our study, 50 μmol/kg NaHS seems to be the most protective dose against CP-induced hepatic injury. In fact, there is a need for further studies to determine the therapeutic range of doses.

## Figures and Tables

**Table 1.** Effects of NaHS Treatment on Liver IL-6, IL-10, cGMP, NO, H_2_S Levels in Rats With CP-Induced Hepatotoxicity

	Control	CP	NaHS25	NaHS50	NaHS100
**IL-6** pg mg^−1^ total protein	299.40 ± 27.69	485.58 ± 18.91^***^	395.84 ± 10.46**^x^** ^**^	301.70 ± 15.57**^xxx&&^**	431.22 ± 10.27**^+++^** ^***^
**IL-10** pg mg^−1^ total protein	182.60 ± 20.39	401.68 ± 26.99	323.45 ± 14.98**^x^** ^***^	240.06 ± 13.94**^&^** **^xxx^**	372.45 ± 13.32**^+++^** ^***^
**cGMP** pmol. mg^−1^ total protein	84.89 ± 4.34	41.59 ± 4.13^***^	51.23 ± 4.07^***^	62.29 ± 3.25**^xx^** ^**^	48.24 ± 3.19**^++^** ^***^
**NO** µmol mg^−1^ total protein	0.049 ± 0.005	0.016 ± 0.002	0.029 ± 0.002**^x^** ^***^	0.039 ± 0.002**^xxx^**	0.022 ± 0.001**^++^** ^***^
**H** **_2_** **S** µmol g^−o^ total protein	1.30 ± 0.18	0.93 ± 0.28	1.10 ± 0.06**^##^**	1.26 ± 0.13**^#^**	2.06 ± 0.22**^xx^** ^*^

Data are given as mean ± SEM. *P < .05, **P < .01, ***P < .001 vs. control group, ^x^ *P* ˂˂.05, ^xx^ *P *< .01, ^xxx^ *P *< .001 vs. CP group, ^&^ *P *< .05, ^&&^ *P *< .01, ^&&&^ *P* < .001 vs. NaHS25 group, ^+^ *P* < .05, ^++^ *P *< .01, ^+++^ *P* < .001 vs. NaHS50 group, ^#^ *P* < .05, ^##^ *P* < .01, ^###^ *P* < .001 vs. NaHS100 group. ANOVA (n = 8). ANOVA, analysis of variance; C; Control CP; Cyclophosphamide, NaHS; Sodium hydrosulfide; SEM, standard error of mean.
